# Decompression Illness in Repetitive Breath-Hold Diving: Why Ischemic Lesions Involve the Brain?

**DOI:** 10.3389/fphys.2021.711850

**Published:** 2021-09-03

**Authors:** Kiyotaka Kohshi, Petar J. Denoble, Hideki Tamaki, Yoshitaka Morimatsu, Tatsuya Ishitake, Frédéric Lemaître

**Affiliations:** ^1^Division of Neurosurgery, Nishinihon Hospital, Kumamoto, Japan; ^2^Department of Environmental Medicine, Kurume University School of Medicine, Kurume, Japan; ^3^Divers Alert Network, Durham, NC, United States; ^4^Division of Surgery and General Medicine, Tamaki Hospital, Hagi, Japan; ^5^Faculty of Sport Sciences, University of Rouen, Mont-Saint-Aignan, France; ^6^CRIOBE USR 3278, CNRS-EPHE-UPVD, PSL, Moorea, France

**Keywords:** bubbles, AMA, stroke, cerebral infarct, mechanism

## Abstract

Nitrogen (N_2_) accumulation in the blood and tissues can occur due to breath-hold (BH) diving. Post-dive venous gas emboli have been documented in commercial BH divers (Ama) after repetitive dives with short surface intervals. Hence, BH diving can theoretically cause decompression illness (DCI). “Taravana,” the diving syndrome described in Polynesian pearl divers by Cross in the 1960s, is likely DCI. It manifests mainly with cerebral involvements, especially stroke-like brain attacks with the spinal cord spared. Neuroradiological studies on Ama divers showed symptomatic and asymptomatic ischemic lesions in the cerebral cortex, subcortex, basal ganglia, brainstem, and cerebellum. These lesions localized in the external watershed areas and deep perforating arteries are compatible with cerebral arterial gas embolism. The underlying mechanisms remain to be elucidated. We consider that the most plausible mechanisms are arterialized venous gas bubbles passing through the lungs, bubbles mixed with thrombi occlude cerebral arteries and then expand from N_2_ influx from the occluded arteries and the brain. The first aid normobaric oxygen appears beneficial. DCI prevention strategy includes avoiding long-lasting repetitive dives for more than several hours, prolonging the surface intervals. This article provides an overview of clinical manifestations of DCI following repetitive BH dives and discusses possible mechanisms based on clinical and neuroimaging studies.

## Introduction

Underwater breath-hold (BH) diving is practiced casually by millions of beachgoers and snorkelers. Risks of casual BH diving involve ear and sinus barotrauma, shallow water black-out, and drowning but not decompression sickness (DCS) because of limited depth and time involved. However, the extreme BH diving exposure as seen in professional harvester divers, competitive spearfishermen, and freedivers are sometimes associated with acute brain injuries, and in some cases, with chronic dysfunction. There are also some reports about asymptomatic brain lesions in BH divers without a history of acute neurological symptoms. The causes and mechanisms of neurological post-dive conditions are not clear yet, and the term BH diving neurologic deficit (BHDND) was suggested in a symposium (Table 1; Wong, 2006). The stroke-like manifestations and time relation to BH diving in acute cases are suggestive of DCS, cerebral arterial gas embolism (CAGE), and hypoxic brain injury as the most likely causes. Imaging of brains in BHDND reveals lesions similar to stroke and micro-stroke caused by embolism of various origins. While this does not exclude hypoxic injury due to hypoxemia or hemodynamic hypoperfusion, it shifts the focus of discussion toward various forms of decompression illness (DCI), including paradoxical venous gas emboli (VGE), CAGE, and intraarterial growth of bubbles from nanobubble buds on hydrophobic vascular surfaces.

**Table 1:** Clinical manifestations of decompression illness (DCI) in compressed-air and breath-hold (BH) divers.

	Breath-hold divers (Wong, 2006)	Compressed-air divers (Vann et al., 2011)
Non-neurological	Dizziness/vertigo Constitutional^*^ Pain Cardiovascular	Pain Constitutional Dizziness/vertigo Cutaneous Muscle discomfort Pulmonary Auditory Lymphatic Cardiovascular
Neurological	Motor weakness Numbness/paresthesia Consciousness Mental status Altered speech Visual disturbance Coordination Convulsions Bladder/bowel	Numbness/paresthesia Motor weakness Mental status Coordination Consciousness Bladder/bowel

* Constitutional symptoms include headache, fatigue, agitation, nausea, and vomiting.

After 25years of studying dive-related brain injuries in Ama divers, we find that the evidence, although not yet unequivocal, points toward the DCI as a primary cause. This paper aims to review the epidemiology, clinical manifestation, imaging, functional studies, and possible brain injury mechanisms in BH divers, especially Japanese Ama divers. Available data pertain to several distinctive groups, including Polynesian indigenous harvesters, spear-fisher, and competitive freedivers.

## Diving Practices of Ama

Polynesian harvester divers may have the longest history of BH diving, extending into the 20th century. They have used googles and no other equipment or protective suit, but some could dive deeper than 30m and stay underwater longer than 5min. They dived repeatedly for several hours a day with a variable surface time between descents. At the end of the day, divers sometimes manifested neurological symptoms usually of short duration, which became known as “taravana”(Cross, 1965).

Commercial or professional BH divers of Japan and Korea, known in the scientific literature as “Ama” (men and women of the sea) have been in existence for more than 2,000years (Teruoka, 1932). One theory contends that this diving tradition originated from Polynesian pearl divers (Hong and Rahn, 1967). In Japan, Ama divers start their profession at the age of 15–16years and continue working for more than 20years. Divers older than 60years are not rare (Hong and Rahn, 1967; Shiraki et al., 1985).

**Figure 1:**
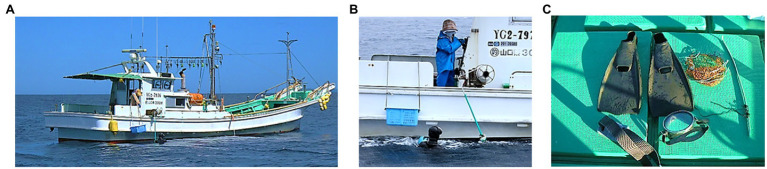
An assisted male Ama diver, called Funado needs his partner (generally, wife) to operate fishing boat, which has a rolling machine to pull up his weight and a basket for seafoods **(A)**. His wife on the boat pulls up an iron-weight and gets closer to Ama after each his dive **(B)**. Ama’s diving equipment includes weight belt, fins, goagle, and hook and net for sea foods **(C)**.

**Figure 2:**
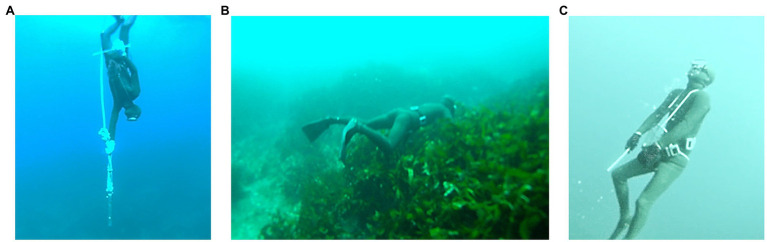
Photos of an assisted male Ama diver during breath-hold diving. He uses an iron weight of 20-kg to descend passively to a depth of 10–30m **(A)**, and he searches and gathers sea foods on the bottom **(B)**. After 1min dive, he swims to the surface without assistance **(C)**.

Ama divers use one of two primary methods: Cachido unassisted diving and Funado assisted diving. Ama divers usually begin their carrier as Cachido divers and dive without any aids to depths of 3–10m. With the experience, they may graduate to Funado and, using weight for faster descent, dive deeper – occasionally over 30m (Figure 1). Funado divers hyperventilate and emit a pursed-lip whistle before descending to the bottom, spending 15–45s for harvesting (Figure 2). They usually stay at the surface 30–60s between the two dives. They work 2–6h a day, usually in two shifts, with a short break for lunch (Tamaki et al., 2010a; Lemaître et al., 2014). In Japan, local unions regulate harvesting season, daily shifts, and diving patterns and do not allow the use of wetsuits to protect their natural resources from over-harvesting in some areas.

Sport spearfishers in the Mediterranean regularly use submarine scooters to achieve quick descents and ascents. They may do 15–20 dives per hour for 3–8h, in a depth range of 20–60m lasting for 2min or more each, with surface intervals in between sometimes as short as 2min or less (Batle, 1999). Freedivers can dive to depths greater than 100m and stay up to 5min and ascend fast to the surface. They practice glossopharyngeal insufflation and exsufflation to increase their diving capacities, which exposes them to additional risks (Lemaître et al., 2009; Schipke et al., 2019).

## Clinical Manifestations of BH Diving Neurological Syndrome

### Post-dive Neurological Manifestation in Ama Divers

Commercial breath-hold divers Ama of Japan and Korea have been studied extensively. A survey among Ama conducted in one diving village in Japan showed that nine out of 16 Funado divers had histories of neurological accidents during or immediately after repetitive BH diving (Kohshi et al., 2001). This prompted a much broader survey which showed that 12 of 173 Ama divers (6.9%) had experienced post-dive stroke-like neurological events (Table 2). The incidence was much higher in Funado (11 out of 29) than in Cachido divers (one out of 144). All affected divers were males (Tamaki et al., 2010a). The most common symptoms were sensory numbness in eight cases, and hemiparesis in six cases. Other symptoms were dizziness, vertigo, nausea, and limb pain. Dizziness was particularly common after continuous long-lasting dives in Funado divers. However, although explicitly asked, Ama divers never reported symptoms suggesting pulmonary barotrauma, like chest pain, hemoptysis, or dyspnea. Two of 12 divers with neurological events also had severe knee and limb pain, but none had a skin rash nor swelling. In 10 of these divers neurological disorders wholly resolved without any treatments, one diver had a residual partial visual deficit, and another had a sensory numbness of the hand. All instances of neurological conditions appeared to be involving the brain. There was no apparent spinal cord involvement, frequently seen in compressed-air diving injuries.

**Table 2:** Diving events in different types of BH divers.

	Spanish spearfishermen (25 cases; Batle, 1999)	Japanese Ama divers (12 cases; Tamaki et al., 2010a)	Competitive athletes (four cases; Tetzlaff et al., 2017)
Manifestation	Constitutional (23)^*^ Numbness/paresthesia (17) Consciousness (13) Motor weakness (11) Visual disturbance (10) Altered speech (7) Dizziness/vertigo (5) Coordination (5) Memory loss (4) Convulsions (1) Sphincter relaxation (1) Auditory disturbance (1) Cardiorespiratory arrest (1) Pain (1)	Dizziness/vertigo (8) Numbness/paresthesia (8) Motor weakness (6) Altered speech (3) Constitutional (2) Pain (2) Visual disturbance (1)	Numbness/paresthesia (3) Motor weakness (2) Altered speech (2) Dizziness/vertigo (1) Unconsciousness (1)

* Parenthesis means total number of cases.

Symptoms in Ama divers were similar to those in Polynesian divers. The stroke-like brain insults appeared during or few minutes after ending several hour-day shifts of repetitive diving to a depth exceeding 20m. Dizziness or blurred vision sometimes preceded the onset of other neurological symptoms (Wong, 2006). The symptoms usually start as mild and may resolve in half an hour or deteriorate within a few hours (Kohshi et al., 1998, 2000, 2020; Tamaki et al., 2010b). One of the clinical characteristics of DCI in Ama divers is that severe neurological symptoms, unlike in CAGE following pulmonary barotrauma, rarely occur suddenly.

### Post-dive Neurological Manifestation in Other Repetitive BH Divers

Batle (1999) reported 25 cases of spearfishers with neurological symptoms appearing immediately on surfacing (Table 2). All divers received recompression therapy and their symptoms entirely resolved. Wong (2006) reported eight cases of Australian BH spearfisher with similar symptoms, short duration, and no sequelae in most cases.

### Post-dive Neurological Manifestation in Single Deep BH Dives

Single deep BH dives following a few shallow dives can cause DCI-like insults, though the cases are less frequently reported. Tetzlaff et al. (2017) summarized reports of stroke-like symptoms following single deep BH dives, including one case they treated and three reported by others. In all four cases, diving depths exceeded 100m (Table 2). The symptoms included motor weakness, sensory numbness, unconsciousness, and speaking difficulty. The case they treated was a 31-year-old man who did three dives to a depth of 100m with 15min of surface intervals and developed speaking difficulty and right-sided motor weakness immediately after the last dive. His laboratory studies and chest CT findings were normal.

### Mental Disorders in BH Diving

Male Ama divers have reported no psychiatric disorders following diving work, although, they may occasionally complain of anxiety during deep and long-lasting dives. In contrast, female Ama divers have suffered specific psychiatric disorders called “Chiyamai” related to their dives on an island (Tochimoto et al., 1998). A survey of 44 female, Ama divers noted that nine of them had mental disturbances related to anxiety attacks. On this island, their diving depths and durations were deeper and longer in other areas (Hong et al., 1991; Mohri et al., 1995). Their diving patterns were similar to those of male Ama divers with diving accidents (Tamaki et al., 2010a). Although, the clinical features of psychiatric disease closely resemble those of some types of panic disorders, female divers did not have depersonalization or de-realization. The clinical symptoms included palpitation, dizziness or unsteady feelings, dyspnea, nausea, and hot flushes; palpitation was the most frequent (Tochimoto et al., 1998). Several Ama divers who had experienced the disorder could not dive and had to stop their diving work. While female Ama divers may have recovered from the disorder, they could not dive at great depths and always had to take anti-anxiety medicine prior to diving. No diving-related psychiatric disturbances have been reported among female or male Ama who dive shallower, in contrast, Polynesian pearl divers frequently felt mental anguish as a form of “taravana” syndrome. A few of them were mentally affected with such symptoms as restlessness, irritability, and poor understanding (Cross, 1965). Mental disorders associated with deep and long-lasting repetitive BH dives are rare, and there is no evidence that their causes are organic.

### Neuropsychological Examinations in BH Diving

Results of neuropsychological studies in BH divers vary. We will mention here only two illustrative studies. Ridgway and McFarland (2006) evaluated neuropsychological investigation in 21 elite freedivers and found no significant differences between the different diving careers. Another study comparing trained BH divers with matched controlled subjects found that divers had slower responses on a Stroop test and more errors on interference card tests (Billaut et al., 2018). These findings were correlated with the maximal BH abilities (*r*=0.73, *p*<0.05) and years of training (*r*=0.79, *p*<0.001), collectively suggesting that apnea training can cause persistent episodic memory impairments.

## Neuroimaging Studies

**Figure 3:**
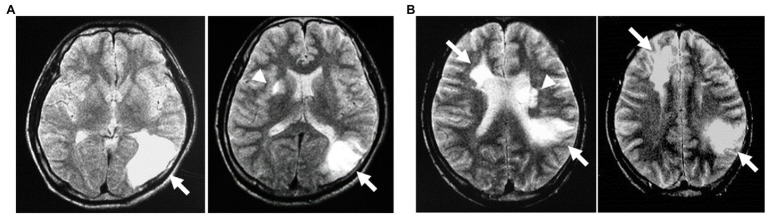
MRI of the brain in a 33-year-old assisted Ama diver with right homonymous hemianopsia. T2-weighted MRI obtained on the 4th day after the diving event shows two increased signal intensities in the left occipital lobe (arrow) and the right basal ganglia (arrow head; **A**). A 39-year-old assisted male Ama diver with right-sided hemiparesis and hemisensory numbness. He suffered from transient left hemiparesis at the age of 17, 25, and 27years, and his T2-weighted MRI on the 3rd day after the event shows three increased signal intensities in the right frontal lobe and left parietal lobe (arrows) and the left basal ganglia (arrow head; **B**; taken from Kohshi et al., 2000 with permission).

We first reported cerebral infarction in Ama divers occurring after repetitive dives to the depths of 15–25m (Kohshi et al., 1998). Since then, we documented by MRI many more cases (Kohshi et al., 2000, 2020; Tamaki et al., 2010b). In Ama divers with post-breath-hold stroke-like symptoms, brain MRIs showed single or multiple cerebral infarcts in areas corresponding to the symptoms and elicited signs. The brain lesions are localized in the cerebral cortex, subcortex, basal ganglia, brainstem, and cerebellum (Figure 3). The neuroradiological findings are consistent with the vascular pathogenesis of the lesions; the presence of cerebral infarcts suggests a shower of various sized emboli migrating into the watershed areas and distal vascular territories of deep perforating arteries (Wodarz, 1980; Mull et al., 1997).

Watershed infarcts are classified into two broad categories as external (cortical) and internal (subcortical) infarcts (Momjian-Mayor and Baron, 2005; Mangla et al., 2011). The formers involve the junction of the distal fields of cortical arteries and are usually wedge-shaped or ovoid, and they may be embolic rather than hemodynamic in nature. The latter are located at the junctions of the cortical arterial territories with deep perforating arteries, showing the rosary-like pattern in the centrum semiovale; they are mainly affected by hypoperfusion due to arterial stenosis or hemodynamic impairment. The most common cerebral lesions revealed by MRI in Ama divers with BHDND appear external watershed infarcts suggesting arterial embolism.

The second group of common lesions seems like lacunar infarcts in the territories of the deep perforating arteries in the basal ganglia and brainstem. The occlusions causing lacunar infarcts are considered to be due to atheromatous changes, secondary hypertension, or diabetes mellitus. Still, one-third may include emboli from cardiac or carotid sources (Horowitz et al., 1992). Lacunar lesions were also reported in compressed gas divers with or without a known history of DCI (Palmer et al., 1992).

The third group of lesions in Ama divers involves the cerebellum (Tamaki et al., 2010b), located in the watershed area between the penetrating cerebellar arterial branches (Savoiardo et al., 1987). Generally, cerebellar infarcts are uncommon in strokes, and more than half of the cases originate from cardiac embolic sources (Kase et al., 1993). In summary, neuroimaging findings in Ama divers with BHDND support the possible role of DCI emboli affecting the external watershed areas (arrows, Figure 3) and the territories of deep perforating arteries of the brain (arrowheads, Figure 3).

In a radiological study of Ama divers without neurological deficits, we found brain changes in 11 out of 12 divers. The age of divers was 44 to 61 (median: 56), and four of them had a history of BHDND (Kohshi et al., 2014). Brain changes in divers without a history of BHDND may be due to the cumulative effects of repeated transient ischemic injury. Even in the absence of MRI signs, single-photon emission tomography (SPECT) in five elite BH divers found diffuse abnormal perfusion of the brain (Potkin and Uszler, 2006). Recently, Moir et al. (2019) have suggested a possible impairment of cerebral autoregulation in elite competitive BH divers. Long-term repetitive BH diving probably affects perfusion and causes local or diffuse cerebral ischemia, and it deserves further studies.

## Possible Mechanisms of DCI in BH Diving

While the signs and symptoms of acute BHDND appear consistent with DCI there are still unresolved questions of underlying mechanisms (Schipke and Tetzlaff, 2016). In BHDND, neuroradiological imaging shows large cerebral infarcts like those in CAGE following pulmonary barotrauma or iatrogenic embolism (Wityk et al., 2001; Gao et al., 2009), but the clinical course is different. The post-dive neurological symptoms in commercial BH divers appear mild at onset and may gradually deteriorate within several hours (Kohshi et al., 1998, 2000; Tamaki et al., 2010b; Cortegiani et al., 2013), while in the CAGE symptoms occur all at once immediately after surfacing (Neuman, 2003). Prompt symptoms resolution with hyperbaric oxygen (HBO_2_) treatment (Paulev, 1965; Tetzlaff et al., 2017) or oxygen (O_2_) inhalation alone (Gempp and Blatteau, 2006) may support the hypothesis that brain insults in BH divers are caused by inert gas bubbles in the brain. However, Ama divers with acute BHDND have neither symptoms nor signs of pulmonary barotrauma, and CAGE appears less likely. It may instead be DCS, but intravascular bubbles in BH divers are rarely detected. Thus, the question of what could cause the cerebral insults in BH divers remains an unresolved issue.

### Nitrogen Accumulation

Direct measurement of nitrogen (N_2_) in peripheral venous blood of unassisted Korean Ama divers after a series of diving to 3–6m has shown a significant but insufficient accumulation to cause DCS (Radermacher et al., 1992). However, N_2_ accumulation could amount to levels necessary to cause DCI in multiple deeper and longer-lasting BH dives with short intervals.

Calculations of saturation and desaturation using compressed gas diving models show that conditions for DCS could occur with deep, repetitive BH dives and short surface intervals (Paulev, 1965; Olszowka and Rahn, 1987). Based on these calculations, it appeared that repeated BH diving to less than 20m would be safe if the surface time is equal to or longer than the dive time (Lanphier, 1965). However, our interview survey found that some Ama divers experienced neurological events after a series of dives to a depth of around 15m and a surface time to a dive time ratio of 0.4 (Tamaki et al., 2010a). Gempp and Blatteau (2006) reported a case of a 21-year-old man who had transient neurological disorders 2h after 10–12 times of BH dives to 10–18m over 60–90min; the diving time was 1–2min each with surface intervals of 5–6min. BHDND after dives predicted safe by models may still be the DCS, but the compressed gas models do not fit BH diving. Neurological problems were also reported in shallow (6–8m) BH diving for more than 4h (Wong, 2000, personal communication), which seems too shallow for DCS.

Single BH dive does not provide enough N_2_ to cause DCS (Paulev, 1965; Olszowka and Rahn, 1987; Radermacher et al., 1992), unless extremely deep and preceded with few shallower preparatory dives. Tetzlaff et al. (2017) presented four cases of competitive freediving athletes experiencing stroke-like insults after BH dives to maximum depths of 100–249.5m. Such dives have been mathematically shown to create supersaturation and generate gas bubbles.

### Detectable Venous Gas Emboli

After repetitive BH dives, N_2_ bubbles are probably formed in the small veins of tissues and flow into the right atrium. Spencer and Okino (1972) used continuous-wave Doppler. They described the signals for 1hour, suggesting air bubbles in a Japanese Ama diver after a 51-min period of 30 dives to 15m in depth. Boussuges et al. (1997), however, using wave Doppler and two-dimensional echocardiography, did not find any circulating bubbles in 10 BH divers for a mean duration of 4h and 3min (2–6h) and at a mean maximum depth of 35m (24–40m). Lemaître et al. (2014) recorded the “lowest” intravascular bubbles (Spencer’s grade 1; occasional bubbles) by precordial Doppler monitoring in only one of 12 Ama divers (mean age: 55.6, 48–66) after 186min of 99 repetitive dives; mean maximal depth and dive duration were 15.8m and 68s, respectively. The bubbles were identified after 2min of detection and lasted only for 10s. While out of these 12 Ama divers, four had previously experienced neurological diving events, ischemic cerebral lesions were found by MRI in 11 of them (Kohshi et al., 2014). Huggins and Stepanek (2006) followed a competitive freediver during 4 consecutive days of training dives with consistent dive profiles up to 50m depth. Only on one occasion, at the end of the day, when the diver overused his right hand, they detected Grade 1 bubbles in his right subclavian vein. Remarkably, this diver did his preparatory dives on functional residual capacity, meaning that the availability of N_2_ in alveoli was minimal. The only impressively high and lasting bubble grade (Eftedal-Brubakk’s grade 4; continuous bubbling) after BH diving was reported in a spearfisher performing 15 training dives in a deep pool with a water temperature of 33°C. The median depth of dive was 40.2m (6.2–41.7m), and the mean duration 140.9s. Interestingly, the computer calculated gradient factor at the end of the dive session was 0.33, usually not associated with such a high bubble grade in scuba diving (Cialoni et al., 2016). The diver’s BMI was 24, which is similar to Ama divers’ (Lemaître et al., 2014). In another recent echocardiography study, Barak et al. (2020) recorded the “lowest” venous bubbles in six out of 11 BH divers for 15min after dives at rest or after provocation. Interestingly, the results were similar after eight dives to 35m for 1h and after 6h of competitive dives to depths between 15 and 40m.

So far, detectable VGEs in BH divers are rare, and it appears that there are more divers with cerebral symptoms than with VGE. However, the measurement results of such bubble detection may simply mean that we have no data of the best measurable condition under which VGEs are the most detectable in BH divers.

On the other hand, in compressed-air diving, despite the abundance of VGE often detected (Nishi et al., 2003), DCS is rare, and spinal involvement is more common than cerebral (Vann et al., 2011). The risk of VGE causing DCS, in general, is low because the pulmonary circulation effectively filters VGE. Why lesions in BH diving mainly involve the brain is an unresolved dilemma. A possibility that BH may generate undetectable microbubbles cannot be excluded (Nishi et al., 2003; Eftedal et al., 2007), despite recent improvement in the resolution of VGE detection tools (Le et al., 2021). VGE in BH diving deserves more systematic studies. Gas embolization alone may not be sufficient to cause DCI. Repeated bubble insults and involvement of other processes may be necessary (Thalmann, 2003; Balestra et al., 2019; Barak et al., 2020).

### Microbubbles and Nanobubbles

Mammalian lungs usually constitute a complete filter for bubbles larger than 21 μ in diameter (Butler and Hills, 1979; Butler and Katz, 1988). Smaller bubbles would not usually cause detectable brain lesions since they can pass through the capillaries of the brain. In an experimental study, Hills and James (1991) showed that such bubbles could transiently impair the blood-brain barrier. One could assume that a prolonged and repeated release of microbubbles might cause permanent damage to the brain. Moreover, gas bubbles serve as an interface for aggregating blood components such as platelets and leukocytes acting as emboli (Francis and Mitchell, 2003).

An interesting theory of decompression bubbles developing from gas micronuclei called nanobubbles has been published recently. The nanobubbles have been shown to occur on active hydrophobic spots. They may also appear on endothelial surfaces in the lungs, pass through intrapulmonary shunts or the patent foramen ovale (PFO), and lodge in remote tissues where they continue to grow. Nanobubbles may also appear as autochthonous bubbles in distal small arteries or capillaries of the brain. With further growth, they can cause cerebral blood flow disorder (Goldman and Solano-Altamirano, 2015; Arieli and Marmur, 2017; Arieli, 2019a,b). While this hypothesis is supported by *ex vivo* experiments (Le et al., 2021), there is no experimental evidence that it happens *in vivo*, and there are also some concerns regarding its’ basic assumptions (Doolette, 2019).

The nanobubbles hypothesis implies multiple small infarcts in the internal watershed areas most vulnerable to hemodynamic impairment (Moody et al., 1990; Momjian-Mayor and Baron, 2005). However, the cerebral lesions in Ama divers with neurological DCI are not situated in these areas (Kohshi et al., 1998, 2000, 2020; Tamaki et al., 2010b), but instead in the external watershed areas (Matsuo et al., 2012).

### Intracardiac and Intrapulmonary Shunts

Gas bubbles formed in the venous blood after long-lasting repetitive BH dives can cross from the venous side to the arterial side (arterialization) through intracardiac or intrapulmonary right-to-left shunt (RLS). The intracardiac RLS, including the PFO and atrial septal deficit, are present in 10–30% of healthy adults (Hagen et al., 1984; Lynch et al., 1984). Bubbles that pass through these shunts can cause DCI as a paradoxical embolization of the brain. In compressed-air divers, the proportion of cerebral ischemic lesions was closely related to intracardiac RLS presence (Knauth et al., 1997; Gempp et al., 2010). The RLS has been documented in at least one BH divers with post-dive DCS-like neurological symptoms (Gempp and Blatteau, 2006), but it has not been detected in Ama divers with the BHDND (Kohshi et al., 2000; Matsuo et al., 2012). These reported cases suggest that neurological DCI in BH divers cannot be explained only by intracardiac RLS and that several possible mechanisms may be involved in cerebral DCI (Fitz-Clarke, 2018). Another pathway allowing blood flow to bypass the lung capillaries are intrapulmonary arteriovenous anastomoses (IPAVA), which provide a route for right-to-left transmission of embolus of 25–50 μ in diameter. IPAVA can open during submaximal exercise (Eldridge et al., 2004; Ljubkovic et al., 2012), or even at rest in hypoxic conditions (Laurie et al., 2010). Schipke and Tetzlaff (2016) suggest that hypoxia enhanced IPAVA opening plays a key role in brain damage in BH divers. Barak et al. (2015) have shown in compressed-air divers that exercise-induced IPAVA enables arterialization of VGE but only a few arterial emboli reach cerebral circulation. Regardless, while the risk may be small, possible role of IPAVA in cerebral DCI after BH diving may not be dismissed.

### Pulmonary Barotrauma

Cerebral arterial gas embolism following pulmonary barotrauma remains a possible cause of DCI in BH divers, at least in some cases. It has been reported in a shallow BH undersurface swimming (Harmsen et al., 2015), in fishermen and freedivers. Lungpacking by glossopharyngeal insufflation can cause lung barotrauma (Jacobson et al., 2006), and AGE before descent (Liner and Andersson, 2010). Deep diving, not necessarily to the depth of total lung collapse, may cause lung injury and AGE. Alaimo et al. (2010) reported a case of a 41-year-old diver who, after 5h of repetitive BH diving to 20–24m of depth, developed neurological DCI like symptoms. A brain CT revealed gas bubbles in carotid and left ophthalmic arteries consistent with AGE. Cortegiani et al. (2013) presented a stroke-like case in a 57-year-old BH fishing champion. The dive pattern and the MRI findings of a subcortical hypointense area in the temporal region were suggestive of DCS. Still, the chest CT findings of a ground-glass pattern indicated the lung squeeze, which could be the source of AGE in this case.

However, acute hemoptysis due to pulmonary barotrauma during deep descents in healthy freedivers is not uncommon (Boussuges et al., 1999; Kiyan et al., 2001; Cialoni et al., 2012; Schipke et al., 2019). Ama divers with BHDND had never reported symptoms of pulmonary barotrauma despite a systematic interrogation (Kohshi et al., 2001; Tamaki et al., 2010a), and their chest radiograms in acute cases demonstrated no abnormal shadows (Kohshi et al., 2000, 2020; Tamaki et al., 2010b; Matsuo et al., 2012). Thus, we believe that pulmonary barotrauma and CAGE is less likely cause of stroke-like neurological disorders with large ischemic cerebral lesions in Ama divers.

### Other Factors

The pathophysiology of DCI in BH divers is not clear at all and is probably multifactorial. On the comments for the viewpoint, Foster et al. (2016) discussed some pathogenetic factors including increase in cardiac output or cerebral blood flow, hypercapnia, and hypoxia. In compressed-air diving, other factors like vascular dysfunction, microparticles, and neutrophil activation have been identified as potential contributors (Thom et al., 2015). Recently, Barak et al. (2020) have also described in BH diving that microparticles play an essential role in endothelial dysfunction of the brain. Although, the circulating microparticles could induce cerebral small vessel disease, the microparticle theory may not completely explain the differences in clinical characteristics of DCI between compressed-air and BH diving, nor the number and location of the large cerebral lesions.

A rapid increase in blood pressure, endothelial dysfunction, a transitory cerebral vascular autoregulation dysfunction, and blood-brain barrier breakdown in BH diving (Barak et al., 2015; Balestra et al., 2019; Patrician et al., 2021) may play a similar role as in a reversible cerebral vasoconstriction syndrome (RCVS) and posterior reversible encephalopathy syndrome (PRES) resulting from a cerebral arterial constriction in a context of major inflammatory diseases or injuries (Ducros, 2012; Pilato et al., 2020). However, clinical presentation and imaging in BHDND is different. Endothelial dysfunction has been observed after BH diving (Nossum et al., 2002; Theunissen et al., 2013; Barak et al., 2020), which may stimulate aggregation of blood components and form thrombi in vessels (Francis and Mitchell, 2003). However, acute neuroradiological findings in BHDND indicate the absence of multiple small cortical infarcts (Kohshi et al., 1998, 2000, 2020; Tamaki et al., 2010b; Matsuo et al., 2012), which suggest a typical shower of thrombotic emboli (Wityk et al., 2001).

The formation of cerebral ischemic lesions in BH divers probably requires gas bubble emboli in the main arteries of the brain. In addition, DCS appears a systemic disease that is related to gas bubbles, but its manifestations depend on multiple factors and individual response of the diver (Balestra et al., 2019).

## New Hypothesized Mechanism

The neuroimaging studies show considerably large ischemic lesions in the external watershed areas and the territories of perforating arteries, a pattern seen in embolic brain injuries. However, VGEs after BH diving are detected rarely and in small quantities (Spencer and Okino, 1972; Lemaître et al., 2014; Barak et al., 2020). Thus, we believe that VGE need not be detectable to initiate processes resulting in cerebral ischemic lesions.

They may cause occlusion of cerebral arteries perfusing the external watershed areas and the perforating arteries. Major surface arteries with higher blood flow, branch, and taper into capillaries in the external watershed areas (Mangla et al., 2011); hence, small bubbles may be propelled by hydrostatic forces to reach this region (Dutka et al., 1988) and migrate into small cerebral arteries (average diameter, 30–-60 μ; Dutka, 1985).

**Figure 4:**
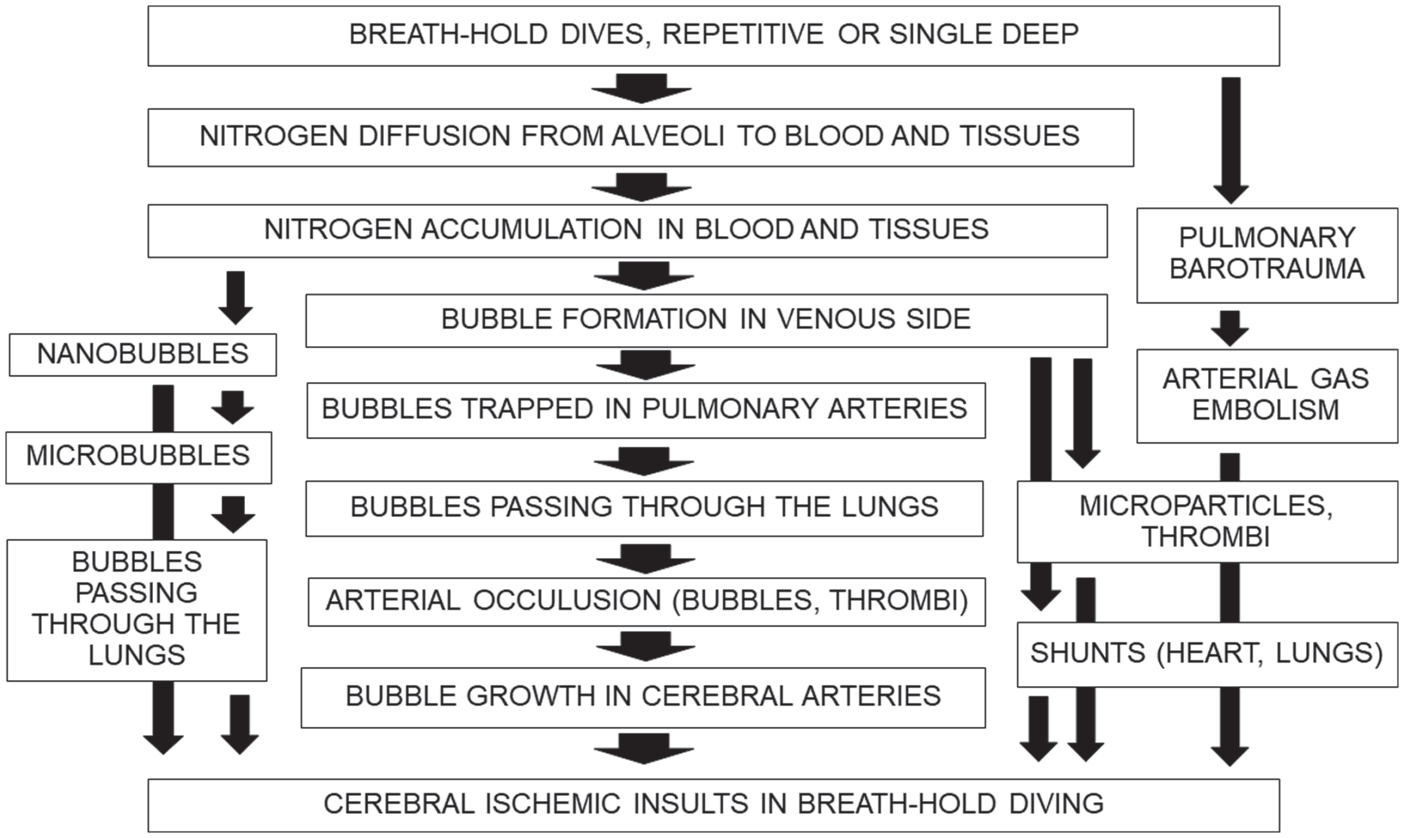
Overview on pathophysiologic mechanisms of DCI in repetitive breath-hold diving. Large arrows show the new hypothesized mechanism and small arrows suggest other possible factors.

These bubble seeds are the first step of neurological DCI in BH divers. Small amounts of intravascular bubble cause endothelial dysfunction (Nossum et al., 2002; Theunissen et al., 2013; Barak et al., 2020), may form thrombi and affect arterial occlusion of the brain. Or microparticles may induce bubble nucleation and contribute to vascular injuries (Thom et al., 2015; Barak et al., 2020). The progressive evolution of neurological disorders in most Ama divers seems compatible with gradual bubble growth. The expansion of bubbles is possible due to N_2_ influx from the end of occluded arteries and surrounding brain tissue (Arieli, 2019a,b). At present, this hypothesis (Figure 4) may explain why cerebral DCI in BH dives, its clinical course, and neuroimaging findings.

## Treatment and Prevention

Experience with the treatment of DCI in BH diving is limited. However, HBO_2_ is the treatment of choice for bubble-related disease. It appears effective for iatrogenic arterial gas embolism in the hyperacute phase (Blanc et al., 2002; Tekle et al., 2013), but not for acute ischemic stroke (Bennett et al., 2014). HBO is very effective in cerebral DCI of compressed gas divers when administered within 6h of symptom onset (Blatteau et al., 2011). Similarly, when administered with a short delay, the HBO is effective in BH diving DCI (Batle, 1999; Wong, 2006; Cortegiani et al., 2013; Tetzlaff et al., 2017).

Breathing normobaric O_2_ immediately upon symptom onset has been beneficial in compressed gas diving DCI (Longphre et al., 2007), and has become a standard first aid in recreational scuba diving.

Treatment of DCI in commercial and competitive BH divers should follow the practices adopted in compressed gas diving. Normobaric O_2_ should be administered immediately upon symptom onset, followed by HBO_2_ therapy as soon as possible. Early treatment may prevent permanent brain injury.

Prevention of DCI is critical for BH divers; the best risk mitigation strategy is to reduce exposure and increase surface times between consecutive dives. While diving depth and duration and bottom time are well known as risk factors for DCI in BH diving, the short surface interval and long diving shifts are probably major causes in BH dives to 10–20m.

Breathing normobaric O_2_ after long-lasting repetitive BH dives appears protective in BH and compressed gas diving (James, 2007; Blatteau and Pontier, 2009; Castagna et al., 2009). However, diving fishermen around the world may have no access to use O_2_ for their diving work. Thus, the best strategy for mitigating the DCI risk in repetitive BH diving remains taking longer surface intervals and limiting the diving shift to less than 2h.

## Conclusion

The BH diving neurological deficit, both acute stroke-like manifestations and asymptomatic lesions, in our opinion, are decompression disorders initiated by gas embolism. Underlying injuries occur in the external watershed areas and the territories of perforating arteries of the brain, an area vulnerable to arterialized venous gas bubbles, which in the conditions of repetitive BH diving can grow inducing processes that lead to DCI. The mechanisms of brain injury following repetitive BH diving are not clear and seem to be multifactorial, but more research is necessary to establish complete understanding.

## Author Contributions

FL, KK, and TI contributed to concept and design of the study. HT, KK, TI, and YM obtained divers’ data (images and clinical history), and FL took all photos. KK wrote the first draft of the manuscript. PD critically reviewed the paper. All authors contributed to the article and approved the submitted version.

## Conflict of Interest

The authors declare that the research was conducted in the absence of any commercial or financial relationships that could be construed as a potential conflict of interest.

## Publisher’s Note

All claims expressed in this article are solely those of the authors and do not necessarily represent those of their affiliated organizations, or those of the publisher, the editors and the reviewers. Any product that may be evaluated in this article, or claim that may be made by its manufacturer, is not guaranteed or endorsed by the publisher.
